# How the Donor/Acceptor Spin States Affect the Electronic Couplings in Molecular Charge-Transfer Processes?

**DOI:** 10.1021/acs.jctc.1c00126

**Published:** 2021-04-08

**Authors:** A. Kubas

**Affiliations:** Institute of Physical Chemistry, Polish Academy of Sciences, Kasprzaka 44/52, 01-224 Warsaw, Poland

## Abstract

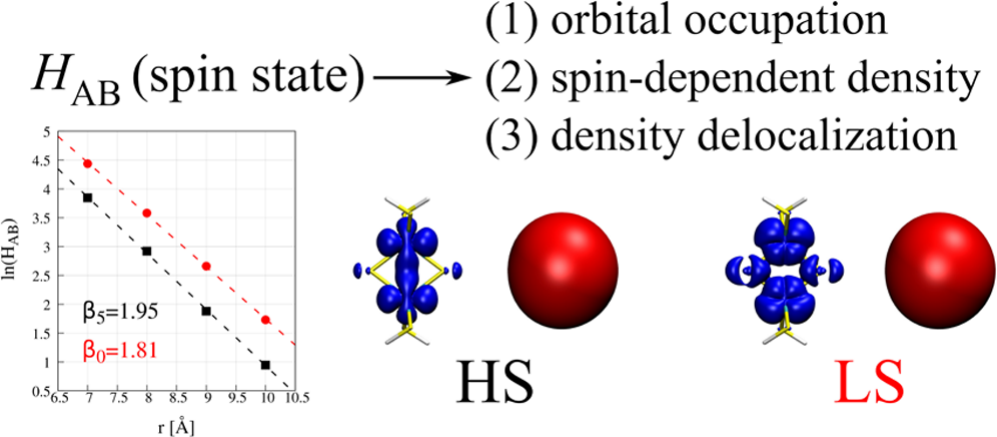

The electronic coupling matrix element *H*_AB_ is an essential ingredient of most electron-transfer theories. *H*_AB_ depends on the overlap between donor and acceptor wave functions and is affected by the involved states’ spin. We classify the spin-state effects into three categories: orbital occupation, spin-dependent electron density, and density delocalization. The orbital occupancy reflects the diverse chemical nature and reactivity of the spin states of interest. The effect of spin-dependent density is related to a more compact electron density cloud at lower spin states due to decreased exchange interactions between electrons. Density delocalization is strongly connected with the covalency concept that increases the spatial extent of the diabatic state’s electron density in specific directions. We illustrate these effects with high-level *ab initio* calculations on model direct donor–acceptor systems relevant to metal oxide materials and biological electron transfer. Obtained results can be used to benchmark existing methods for *H*_AB_ calculations in complicated cases such as spin-crossover materials or antiferromagnetically coupled systems.

## 1 Introduction

Electronic coupling matrix element *H*_AB_, also called resonance or charge-transfer (CT) integral, is a key ingredient of electron-transfer theories. It is defined as
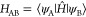
1where *Ĥ* is the electronic Hamiltonian of the system. The diabatic wave functions ψ_A_ and ψ_B_ represent charge-localized states of the donor and acceptor, respectively. According to Fermi’s golden rule, the probability of ET process, hence the ET rate *k*_ET_, is proportional to the square of the *H*_AB_
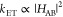
2

In the weak-coupling limit (nonadiabatic case), the ET rate can be calculated within the Marcus theory, where the rate is expressed as a combination of just three system-dependent parameters: the driving force, Δ*G*°, the reorganization free energy, λ, and electronic coupling between the initial and final ET states *H*_AB_(1,2)
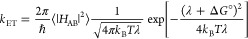
3

As the charge-transfer integral enters with a square to eqs 2 and 3, the accuracy of the final ET rate will critically depend on the accuracy of *H*_AB_ determination. Recently, we provided the community with two databases, HAB11^3^ and HAB7,^4^ of benchmark quality electronic coupling matrix elements for symmetric dimers that are now widely used to test new methods for *H*_AB_ calculations (see, e.g., refs (5−9)). Our findings show clearly that the absolute value of the resonance integral depends on the way the diabatic states are obtained—constrained density functional theory (CDFT)^10−13^ with 50% of exact exchange was found to be an excellent choice that provides couplings close to the reference values. The dependency of the *H*_AB_ on the amount of exact exchange is closely related to the self-interaction error^14^ that plagues approximated density functionals—without the exact exchange, we observed spurious donor electron density delocalization onto the acceptor fragment that increases the coupling significantly. With an increasing percentage of exact exchange, the electron density becomes more compact and the couplings decrease. The delocalization error manifested itself also as a high overlap between donor and acceptor diabatic wave functions.

The dependency of the *H*_AB_ on the overlap integral between the initial and final electronic states is a well-known fact. In the fundamental work on the double exchange, Anderson and Hasegawa^15^ noted that the electron-transfer integral depends on the overlap between magnetic centers. In the extended Hückel theory,^16^ the off-diagonal Hamiltonian matrix elements (resonance integrals) were made proportional to the overlap between atomic orbitals. Schastnev and Luken^17^ used the Linderberg formula^18^ to relate the resonance integral with the derivative of the overlap integral wrt distance between the centers of mass of the charge-localized electron clouds.

Based on these observations, a family of fragment orbital (FO) DFT approaches was derived.^13,19−21^ In FO DFT schemes, the two charge-localized wave functions are optimized in separate calculations. The electronic coupling matrix element is then obtained *via* appropriate orthogonalization using overlap between wave functions. In most practical applications, however, the coupling is considered in a simplified manner, i.e., the donor and acceptor molecular orbitals enter expression 1 in place of donor and acceptor diabatic wave functions, respectively.^22^ Such an approach is well justified when the electron transfer takes place essentially between frontier molecular orbitals. The explicit dependency of the *H*_AB_ on the overlap between donor and acceptor states was explored recently in the development of the analytical overlap method (AOM)^23^ for ultrafast *H*_AB_ estimations.

The works of Petrenko and Stein^24,25^ are rare examples of FO DFT calculations where the wave functions of individual fragments are considered explicitly in the *H*_AB_ computations. The authors calculate electronic coupling matrix elements for the biological ET process that involves iron–sulfur clusters. Teo et al. performed both static DFT^26^ and molecular dynamics simulations^27^ of charge transport between a protein-containing iron–sulfur cluster and the DNA double strain. In any case, a complicated electronic structure of [Fe_4_S_4_] clusters was covered in an approximate way using a broken-symmetry approach to couple individual high-spin (HS) Fe^+2/+3^ centers to overall low-spin (LS), antiferromagnetic states.^28−30^ Here, we should also note earlier works that treat biological ET between iron–sulfur clusters differently without resorting to the FO DFT approach, i.e., using tunneling orbitals of Stuchebrukhov.^31−33^ In all of these works, particular attention was paid to represent the antiferromagnetically coupled systems’ wave function in a physically sound way.

Studies of photoinduced electron transfer often assume the *H*_AB_ for radical ion pair to be very similar in the singlet and triplet cases.^34^ When thinking about the ET process that involves antiferromagnetically coupled systems, it is also tempting to resort to the high-spin wave function as the problem reduces to a single-determinant case. In the context of iron–sulfur clusters, we have shown recently^35^ that high-energy UV–vis spectrum of model [Fe_2_S_2_] complex can be efficiently simulated with the high-spin configuration interaction (CI) approach provided the reference orbitals come from the low-spin spin-averaged restricted open-shell calculations.^36^ Karlström and Malmqvist calculated the *H*_AB_ for diatomic ET in the Fe^2+^–Fe^3+^ system using high-spin orbitals and subsequent nonorthogonal CI calculations.^37^ They noted that *a few test calculations showed that the interaction matrix element (...) was quite insensitive to the total spin* but did not provide data to judge how big the influence really was.

Rosso and co-workers^38−40^ reported a series of complete active space self-consistent field (CASSCF)^41^ calculations on model dimeric motifs found in various open-shell transition-metal oxides. They found that the magnitude of *H*_AB_ in these systems depends on the crystallographic direction, and the difference cannot be explained by merely different donor–acceptor distances. In fact, roughly 5 times smaller couplings were found in the lattice directions that feature antiferromagnetic coupling (low-spin dimers). The results were in line with the experimentally observed anisotropy in the electrical conductivity of the hematite single crystals.^42^ The differences were ascribed to a lower average volume of low-spin metal cations^43^ that results in a smaller overlap between the donor and acceptor.

Iron–sulfur clusters feature an internal antiferromagnetic character, and the ET process in FeS-containing proteins occurs between such systems. If the ET would naively take place between Fe centers only, one would expect the ET process to be ineffectively slow in such a case. Bominaar et al.^44^ have shown that activation energy for self-exchange reactions between iron and sulfur clusters decreases with increasing intramolecular electron delocalization. Such a reduction leads to an enhanced rate constant for electron transfer. Moreover, close-lying excited states may also contribute to an increased rate that is especially important in the context of complicated electronic structures of the iron–sulfur clusters.^45^

Spin-dependent properties are essential for systems that, upon the action of an external stimulus (e.g., temperature, light), can change their spin state, like in the case of spin-crossover (SCO) complexes.^46,47^ Such spin-switchable molecules are emerging species, especially in the context of molecular electronic and spintronic.^48^ The SCO materials display interesting conductivity hysteresis with variable temperatures,^49,50^ where the low-spin system is found to be more conductive (although with some exceptions).^51^ Experiments suggest that the charge transport in these materials takes place by polaron hopping.^50^ The hysteresis is then explained by structural changes to the lattice parameters caused by a spin change (distance between metallic centers or inequal phonon contribution at high- and low-spin states).^51,52^ Calculations using Green’s functions^53,54^ show that the charge transport through the SCO molecule is strongly spin-dependent. It was also shown that when an SCO complex is placed on a metallic surface, high- and low-spin states will display different degrees of charge transfer from the surface.^55^ Moreover, redox properties of SCO molecules are also strongly spin-dependent.^56^

It is therefore clear that the spin states of isolated charge carriers, as well as the spin state of the full quantum system, are important factors that dictate the efficacy of the ET process. However, the spin-state effect has a multidimensional character that needs to be captured in a balanced way by any electronic structure method that aims to provide reliable estimates of the electronic coupling matrix elements for any spin state accessible in the examined system.

In this paper, using various direct donor–acceptor model systems, we discuss three chemically relevant factors that make the electronic coupling matrix element-dependent on the spin state of the system: (a) the orbital occupation, (b) spin-dependent electron density, and (c) density delocalization. The classification is not strict but useful when designing the simulation strategy for real molecular systems. We show under which circumstances the *H*_AB_ will be only marginally spin-dependent. We also demonstrate that the density difference maps between the ground and charge-transfer (CT) states are useful tools in quantifying the spin state’s influence on the absolute value of the electronic coupling matrix element.

## 2 Computational Details

Electronic coupling matrix elements were obtained using the generalized Mulliken–Hush (GMH) method.^57^ The method can be viewed as an approach to transform orthogonal adiabatic ground and CT states to the diabatic basis of charge-localized states. The key assumption here is that the transition moment connecting the diabatic states is zero (μ_AB_ = 0). Briefly, for the 2-state donor–acceptor problem, one can collect adiabatic energies in the following matrix
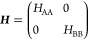
4where *H*_AA_ and *H*_BB_ are the ground- and excited-state energy expectation values associated with the adiabatic, orthogonal wave functions Ψ_A_ and Ψ_B_, respectively. The corresponding dipole moment matrix is defined as
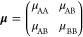
5Here, the state dipole moments μ_AA_ and μ_BB_ are taken in the direction of the transition dipole moment matrix element μ_AB_. We search for the matrix **P** that brings **μ** into the diagonal form **μ**’. In such a case, μ_AB_^′^ = 0. The same transformation can be applied to matrix eq 4 so that the off-diagonal elements will now correspond to the electronic coupling matrix elements *H*_AB_ between charge-localized states
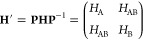
6and
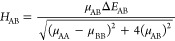
7In eq 7, Δ*E*_AB_ denotes the energy difference between the adiabatic excited and ground states. We note that the method is generally applicable to nonsymmetric systems. However, for symmetric dimers where μ_AA_ = μ_BB_, it reduces to a simple form of
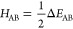
8Thus, all necessary quantities in the GMH method are readily available from the popular quantum chemical codes, and the quality of obtained couplings depends only on the method used for electronic structure calculations of the ground and excited states.

In the present work, electronic structure calculations were performed in multiple steps:

1. Whenever required, the geometries of individual monomers were obtained with the DFT approach using BP86 functional^58^ and def2-TZVP basis sets^59^ augmented with Grimme’s D3BJ dispersion correction,^60,61^
2. We carried out state-averaged CASSCF calculations^41^ in the donor–acceptor (D–A) system at various D–A distances, separately for each spin state of interest; the averaging procedure is described in greater details for each model (Supporting Information provides isosurface plots of active space orbitals along with the leading configuration state functions for all iron-containing systems),
3. If possible, calculations were supplemented with the dynamic correlation treatment either at the CI-level with multireference CI method (MRCI^62,63^ with a simple Davidson correction^64^) or using a perturbative approach with the *n*-electron valence-state perturbation theory (NEVPT2) method,^65−67^
4. We generated state- and spin-specific natural orbitals for each state of interest and computed density difference plots to gain insights into the spatial extent of the involved states,
5. As the *H*_AB_ decays essentially in an exponential way wrt D–A distance *d*, we also obtained the decay constant β for each system studied
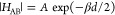
9

Methods used in the present study were extensively tested in our previous work.^3^ MRCI+Q is the most accurate method adapted, followed by the NEVPT2 approach (mean relative unsigned error wrt MRCI+Q of 6.9%^3^). We further confirmed these observations by carrying out electronic coupling matrix element computations for H_2_–H_2_^+^ dimer using the CASSCF method with active spaces of various sizes augmented with different approaches to cover dynamic correlation. The reference couplings were obtained at the full configuration interaction (FCI) level. The results are collected in Table S3 in the Supporting Information. We found excellent agreement between MRCI+Q, NEVPT2, and FCI methods. Even the minimal active space CASSCF method accounted correctly for the coupling difference obtained at high- and low-spin states (relative error of 9.1%).

If not stated otherwise, the ma-def2-TZVPP basis set was used for all elements except hydrogen. In the latter case, either aug-cc-pVTZ basis^68^ was employed (H_2_–H_2_^+^ dimer) or a compact def2-TZVP basis was used in calculations. The aug-cc-pVTZ basis set was shown to provide coupling values that are saturated wrt the basis set.^3^Table S4 in the Supporting Information provides comparison of electronic couplings for the Fe^2+^···Fe^3+^ dimer obtained with ma-def2-TZVPP and aug-cc-pVTZ basis sets. We found excellent agreement between the two sets with ma-def2-TZVPP providing couplings with a mean absolute error of 1.5 meV and a mean relative error of 2.3% wrt a larger aug-cc-pVTZ basis set.

Whenever possible, Coulomb and exchange integrals were evaluated using a resolution of identity (RI)^69^ and chain of sphere approximations,^70^ respectively, along with the related auxiliary basis set (Aux).^71,72^ If the latter was not available for a given basis/element combination, then the Aux basis was generated automatically.^73^

The computations were carried out using Turbomole 7.4 suite of programs^74^ (DFT geometry optimizations) and ORCA 4.2 program^75^ (all subsequent CASSCF-based calculations). Density differences were generated numerically with Multiwfn 3.7^76^ using cube files obtained with the *orca_plot* utility. Raw couplings for all models studied and geometries of iron clusters can be found in the Supporting Information.

## 3 Results and Discussion

Here, we classify the factors that make the calculated electronic coupling matrix elements spin-dependent into three categories: (a) orbital occupation, (b) spin-dependent electron density, and (c) density delocalization. Each case is illustrated with calculations on the model system.

### 3.1 Orbital Occupation

**Figure 1:**
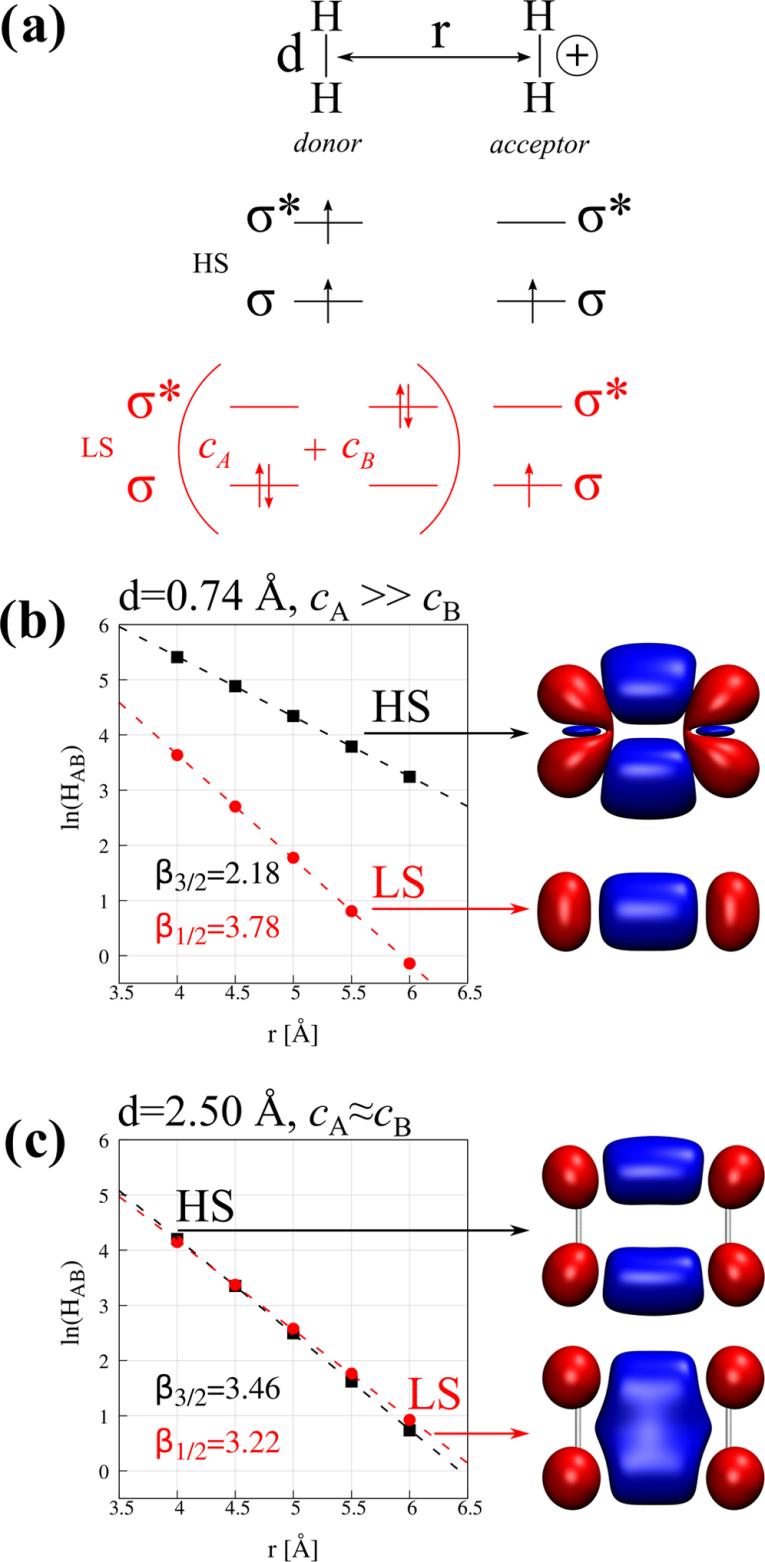
(a) Schematic representation of the model symmetric system for the electron transfer, H_2_···H_2_^+^. Diabatic molecular orbital pictures in the high- (HS) and low-spin (LS) electronic states are provided for the system before electron transfer. Panels (b) and (c) depict the change of the *H*_AB_ with the distance r between donor and acceptor molecules at the equilibrium and elongated H–H distance (*d*), respectively. For each electronic state, the representative difference density plots are provided on the right (±0.0005 au).

The states of different multiplicities often have distinctive orbital occupation patterns so that the orbitals involved in the charge-transfer process are different. This should be reflected in the *H*_AB_ values. Consider, for example, a symmetric H_2_···H_2_^+^ system (see Figure 1a). Here, we calculated the coupling integrals at various intermolecular distances *r* for the two H–H bond lengths, equilibrium *d* = 0.74 Å (Figure 1b) and stretched *d* = 2.50 Å (Figure 1c). In each case, the reference complete active space self-consistent field (CASSCF) wave function^41^ was composed of three electrons distributed over 20 orbitals [CAS(3,20)]. The orbitals that entered the active space were hydrogen’s 1s, 2s, and three 2p atomic orbitals (5 orbitals per H atom). Correlation outside the active space was covered with the MRCI treatment, although in this particular case, the weight of the reference wave function was >0.99 in all cases. For each spin state, a separate set of state-averaged orbitals was obtained. The averaging procedure always involved two states.

For the H–H bond lengths <5 Å, the system has a doublet ground state (low-spin state, LS) and a quartet state (high-spin state, HS) higher in energy. In Figure 1a, the occupations of the idealized, fragment-localized molecular orbitals (diabatic states) of the donor and acceptor molecules in the initial state are shown. In the HS state, the donor is in the triplet state with the two electrons occupying σ and σ* orbitals. In the LS state, the donor is in the singlet state that in general is a linear combination of determinants, each having σ or σ* orbital doubly occupied, with appropriate coefficients *c*_A_ and *c*_B_.

At the equilibrium H–H bond length, the LS state is characterized by *c*_A_ ≈ 1 so that the donor molecule is essentially in a closed-shell configuration and features a doubly occupied σ orbital. Thus, the electron exchange in this electronic state will occur mainly *via* the σ–σ path. On the contrary, spin-conserving charge transfer in the HS state will involve σ* orbitals of both fragments. The couplings for the two electronic states are, as expected, very different (see Figure 1b). *H*_AB_ values in the HS case are more than 5 times larger than those in the LS case. Moreover, the decay constant β is very different in the two cases with 2.18 and 3.78 for HS and LS states, respectively. Inspection of the difference densities in both electronic states confirms that the nature of involved frontier molecular orbitals must differ for the two distinct electronic states. Large differences in the electronic coupling values, as well as dramatically different decay constants, come from the fact that the σ* orbital at the equilibrium distance is much more diffuse than the σ orbital.

The differential spatial extent of the σ–σ* orbitals becomes less pronounced when the H–H bond is stretched. When the bond is set to 2.5 Å, the triplet state of the donor H_2_ molecule is found only 211 meV above the ground singlet state. In the dimer, the LS state is characterized by an almost equal mixture of the two determinants (*c*_A_ ≈ *c*_B_). Here, the electronic couplings at every intermolecular distance *d* are similar along with the decay constant. Decreased repulsion between 1s orbitals makes the spatial extent of the σ* orbital less pronounced. The differential density plots for the two states are quite similar, with the major difference in the system’s inversion center that features increased electron density gain upon excitation. This is due to the interaction between diabatic σ orbitals that have maximum along the H–H axis compared to σ* orbitals that possess a nodal plane along the axis.

### 3.2 Spin-Dependent Electron Density

**Figure 2:**
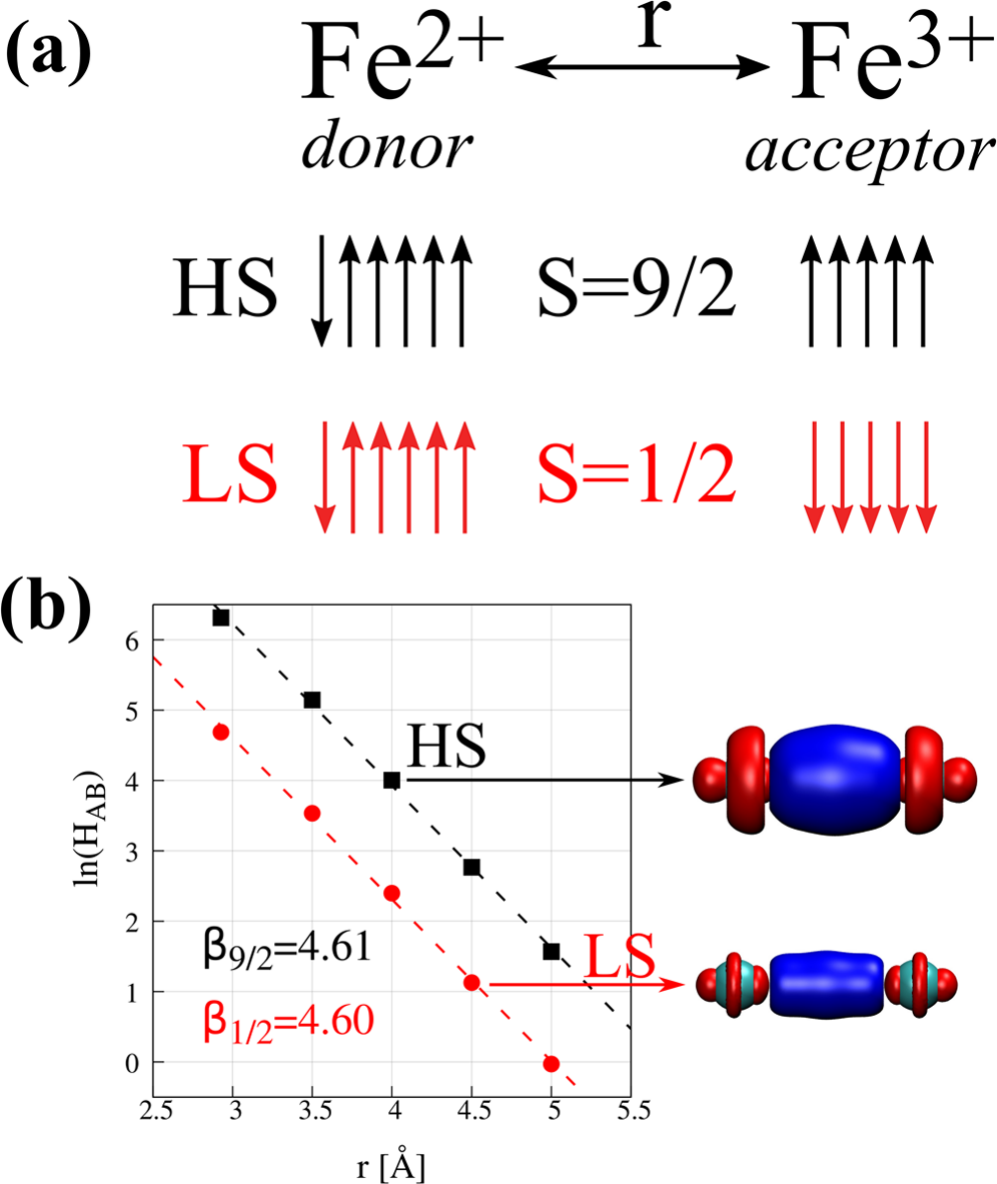
(a) Schematic representation of the model symmetric system for the electron-transfer, Fe^2+^–Fe^3+^ dimer. Diabatic distribution of 3d electrons in the high- (HS) and low-spin (LS) electronic states is provided for the system before electron transfer. Panel (b) depicts the change of the *H*_AB_ with the distance r between donor and acceptor atoms. For each electronic state, the representative difference density plots are provided on the right (±0.0005 au).

The link between orbital occupancy and the electronic coupling matrix element shown in the previous paragraph is rather intuitive. However, it is somehow restricted to situations where the spin state of the donor or acceptor changes separately. Let us now turn our attention to systems where the spin state of the entire quantum system of the donor and acceptor plays a central role, i.e., to the species that feature antiferromagnetic coupling. One of the simplest yet important model systems here is the Fe^2+^–Fe^3+^ dimer shown in Figure 2a. In this case, we set up our CASSCF wave function with all 3d orbitals of both iron centers along with 11 electrons [CAS(11,10)]. The dynamic correlation was accounted for with the NEVPT2 method. We considered two spin states: high spin with *S*_tot_ = 9/2 and low spin with *S*_tot_ = 1/2 denoted as HS and LS, respectively. The state-averaged procedure was performed separately for both spin states, and averaging involved 10 states for each case.

Obtained high-spin couplings are smaller than those obtained by Karlström and Malmqvist^37^ despite the excellent agreement of the β values (4.75 and 4.72 for the earlier and present studies, respectively). For example, at 4.57 Å, our *H*_AB_ equals 13.3 meV at the NEVPT2 level (8.0 meV with reference CASSCF), while 40.0 meV^37^ was obtained using the CAS state-interaction method (CASSI)^77^ using the same active space. Such a discrepancy may originate from different basis sets employed—in the latter case, the decontracted Wachters basis set^78^ augmented with few diffuse functions was used. To check for this possibility, we carried out our calculations with the basis set prepared in the same way as in the previous work. The high-spin coupling at 4.57 Å was found to be 15.7 meV at the NEVPT2 level. Thus, the basis set cannot be responsible for the observed differences. Another suspect is the state-averaging procedure—when we decreased the number of states in the averaging procedure to just two, the coupling increased to 17.5 meV in the Wachters’ basis set, still not enough to account for the discrepancies. As an input into the CASSI procedure, two orbital sets were provided: the first set containing ground-state, state-specific, self-consistently optimized CAS orbitals of the Fe^2+^–Fe^3+^ dimer and the second set generated by appropriate symmetry operations from the first set. Ground-state CASSCF calculations localize the excess electron on one iron site, and such orbitals would be unsuitable for direct excited-state calculations. An appropriate superposition of input states is formed within the CASSI procedure, and configuration interaction (CI) coefficients are optimized. The method may miss some small portion of orbital relaxation upon electron excitation by operating on ground-state orbitals, manifesting itself in slightly higher excitation energies and increased couplings. In our case, ground and excited states are treated on the same footing with the state-averaged procedure, and such a bias is absent. We also note that our couplings are somewhere in between values reported earlier for the same system using the corresponding orbital transformation of the unrestricted Hartree–Fock orbitals expanded in basis sets of various types.^79^

The comparison of calculated couplings for the two spin states does not confirm earlier reports^37^—we found LS couplings to be ca. 5 times smaller as compared to HS couplings (see Figure 2b). A similar trend was observed for electronic couplings in the iron oxide dimers representative for the hematite motifs.^38−40^ Rosso et al.^38,39^ argued that smaller coupling in the LS case is a result of a smaller volume of the low-spin cations that results in the decreased overlap. Furthermore, we note that the effective radii of transition-metal cations decrease upon transition from high-spin to low-spin state.^80^ As a consequence, the coordination bond lengths are shorter for low-spin complexes. Such an effect is widely explored in spin-crossover complexes.^81^

However, the individual iron centers in the Fe^2+^–Fe^3+^ dimer are essentially in the high-spin state. It is the alignment of spins wrt partner center dictated by exchange interactions that makes the system weakly antiferromagnetic—at a distance of 2.9 Å, the antiferromagnetic state is found to be 5 meV lower in energy as compared to a ferromagnetic state. The wave function of the LS state is highly complicated; it also consists of determinants that feature multiple doubly occupied d orbitals. Thus, effective exchange interactions within individual atoms are expected to be lower than for the HS state, which should translate into an effectively decreased ionic radius. Indeed, an inspection of difference densities shows two things (Figure 2b). First, the overall shape for HS and LS states is very similar. Second, the net change caused by the CT excitation is much smaller for the LS state. We interpret this outcome as a manifestation of a smaller overlap between diabatic wave functions.

The variation of HS and LS couplings with the interatomic distance is strikingly parallel. Based on the double-exchange model,^15^ Belinsky^82^ derived a simple expression for the spin dependency of the overlap integral between charge-localized states. For the Fe^2+^–Fe^3+^ dimer where the coupling occurs predominantly *via* atomic d_*z*^2^_ orbitals of both iron centers (denoted as A and B), the overlap *S*_AB_ is expressed as
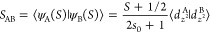
10

In eq 10, *S* is the total spin quantum number for the system, while *s*_0_ is the minimal spin of the paramagnetic ions. The latter is 0 in the present case. The ratio *S*_AB_^*S*=9/2^/*S*_AB_^*S*=1/2^ = 5 and is very close to the ratio *H*_AB_^*S*=9/2^/*H*_AB_^*S*=1/2^ = 5.03 averaged over a set of computed distances.

### 3.3 Density Delocalization

The Fe^2+^–Fe^3+^ dimer is a convenient system that reduces the ET process in a transition-metal-containing species to simple metal-to-metal ET. However, in real materials, incl. oxides or biologically essential ET-mediating cofactors, the surrounding of the metal centers plays a critical role. The nature of ligands dictates the magnetic behavior of the entire complex. Very often, the size of this effect is traced back to the concept of covalency that should be understood according to Solomon et al. as the coefficients of ligand character in the valence metal d-derived molecular orbitals.^83^ Although such a definition is somehow simplistic as it reduces the covalency to a one-electron orbital picture,^84^ the overall effect of increased covalency of a certain metal–ligand bond is the stronger electron delocalization between the coordination pair.

**Figure 3:**
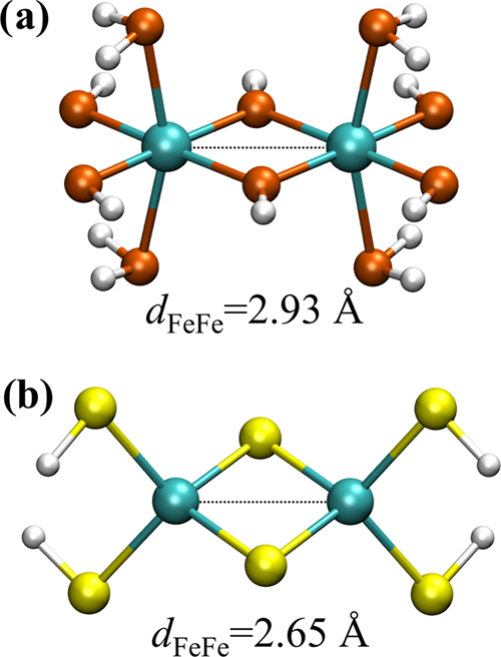
Two model systems used to investigate the effect of covalency on the electronic coupling matrix element calculations: (a) [Fe_2_(OH)_6_(H_2_O)_4_]^0^ that resembles the basic hematite motif (*S*_tot_ = 5 and *D*_2*h*_ symmetry) and (b) [Fe_2_(S)_2_(SH)_4_]^2–^ that models the [2Fe–2S] ferrodoxin active site (symmetry-broken state of *S*_tot_ = 0 and *C*_1_ symmetry).

To find out how electron delocalization, and consequently the covalency, influences relation 10, we constructed two model systems shown in Figure 3: (a) [Fe_2_(OH)_6_(H_2_O)_4_]^0^ that resembles the basic hematite motif similar to one-dimensional (1D) chains used in previous works^79,85^ and (b) [Fe_2_(S)_2_(SH)_4_]^2–^ that models the [2Fe–2S] ferrodoxin active site.^86,87^ Both complexes were optimized with the Fe^3+^ oxidation state of both iron atoms. Complex (a) was assumed to have a total high-spin (*S*_tot_ = 5) and *D*_2*h*_ symmetry. The structure of complex (b) was obtained without symmetry restrictions using a broken-symmetry approach to approximate the true low-spin state observed experimentally for related complexes (*S*_tot_ = 0).^86^ With such fixed geometries, we computed CAS(11,10) wave functions for systems with an excess electron in an analogous way to atomic iron dimer (vide supra). Dynamic correlation outside of the active space was covered in a perturbative way using the NEVPT2 approach.

We expect covalency effects to be large in the iron–sulfur clusters, larger than those found in complexes with oxo-ligands such as the model shown in Figure 3a. Moreover, the covalency of the bond between bridging sulfide ions and Fe centers was more pronounced than bonds involving thiolate ligands.^88^ Consequently, we expect that electron transfer in the [Fe_2_(OH)_6_(H_2_O)_4_]^1–^ model system will resemble the essential physics of the Fe^2+^–Fe^3+^ dimer, while [Fe_2_(S)_2_(SH)_4_]^3–^ may display somehow different behavior. The differences in covalency between the examined molecules are further exemplified by the dramatically different high-spin/low-spin energy gaps of both systems—although both are found to possess an antiferromagnetic low-spin character in the ground state, the high spin is either 0.3 meV or 199 meV above the ground state for oxygen- or sulfur-containing cluster, respectively.

A summary of the electronic coupling matrix element calculations for both models can be found in Table 1. The coupling obtained at the high-spin state for the oxo complex (307 meV) is similar to electronic couplings calculated for 1D iron-oxo chains using various DFT approaches and chains of different lengths (218–265 meV).^79,85^ The ratio of electronic couplings calculated for the hematite model for high- and low-spin states is 5.05, close to the expectation based on eq 10. A similar ratio was obtained at the CASSCF level for closely related iron-oxo dimer considered in the work of Iordanova et al.^39^ On the contrary, the ratio for the iron–sulfur complex is much smaller (1.58), and larger absolute values of couplings were obtained. The latter can be understood by the shorter Fe–Fe interatomic distance along with a high degree of S 3p orbitals mixing with Fe 3d orbitals that enhance the overlap between metallic centers. On the other hand, a departure from the ideal ratio of 5 underlines the role of bridging sulfur atoms in electron-transfer support.

**Table 1:** Electronic Coupling Matrix Elements (meV) for Electron-Transfer Process in Mixed-Valence Model Systems Depicted in Figure 3 Calculated at High- and Low-Spin States (HS and LS, Respectively)

	[Fe_2_(OH)_6_(H_2_O)_4_]^1–^	[Fe_2_(S)_2_(SH)_4_]^3–^
*H*_AB_^HS^	307.3	521.1
*H*_AB_^LS^	60.9	330.1
ratio	5.05	1.58

Molecular motifs shown in Figure 3 often mediate electron transfer from a compatible donor to an appropriate acceptor. For example, in [FeFe]-hydrogenases, the incoming electron flux travels to the hydrogen-evolving active center through a series of iron–sulfur clusters.^89,90^ Hematite is known for its low cost as well as biocompatibility and is a prospective photoanode material that was already coupled to photosystem II.^91^ Therefore, it is of paramount importance to look into the effect of a spin state on the electronic coupling matrix element beyond self-exchange reactions. Here, we investigate the electron transfer from a model source, single calcium atom, to two model systems shown in Figure 3. The choice of calcium was dictated by the energetic compatibility of its 3s orbital with iron 3d orbitals. However, *H*_AB_ value estimations in such systems already put high demands on the electronic structure method.

In this case, the calculations were performed as follows. In the first step, the ground-state wave function featuring all 10 Fe^3+^ 3d orbitals along with 10 electrons was optimized at high- (*S*_tot_ = 5) and low-spin (*S*_tot_ = 0) states yielding two sets of orbitals per system studied. The final orbitals were spit-localized using the Pipek–Mezey method^92^ (active space and doubly occupied orbitals were localized separately). Subsequently, a selected multireference CI procedure was carried out. As a reference, we took 11 configurations that were constructed by allowing single excitations from the calcium 3s orbital into otherwise singly occupied iron 3d manifold. Based on these reference configurations generated in the (12,11) orbital space, single and double excitations were allowed within the active space only. We validated the accuracy of the proposed scheme with *S*_tot_ = 0/*S*_tot_ = 5 state energy splitting in [Fe_2_(S)_2_(SH)_4_]^2–^, which was found to be 1842.1 and 1883.9 cm^–1^ with CASCI and our selected CI approach, respectively.

Canonical CASCI treatment would require more than 1000 states to account for charge-transfer states in the *S*_tot_ = 0 electronic state. The compactness of the minimal active space calculations allowed us to reduce the number of LS states to an absolute minimum that accounts for few first charge-transfer states. In fact, the CI problem had to be solved for 2 and up to 50 states in the case of HS or LS spin states, respectively. The price was that we were unable to augment the calculations with further dynamic correlation treatment outside of the active space. However, the physics behind observed effects will not be altered with any post-CASSCF correction as the orbitals will stay essentially the same.

**Figure 4:**
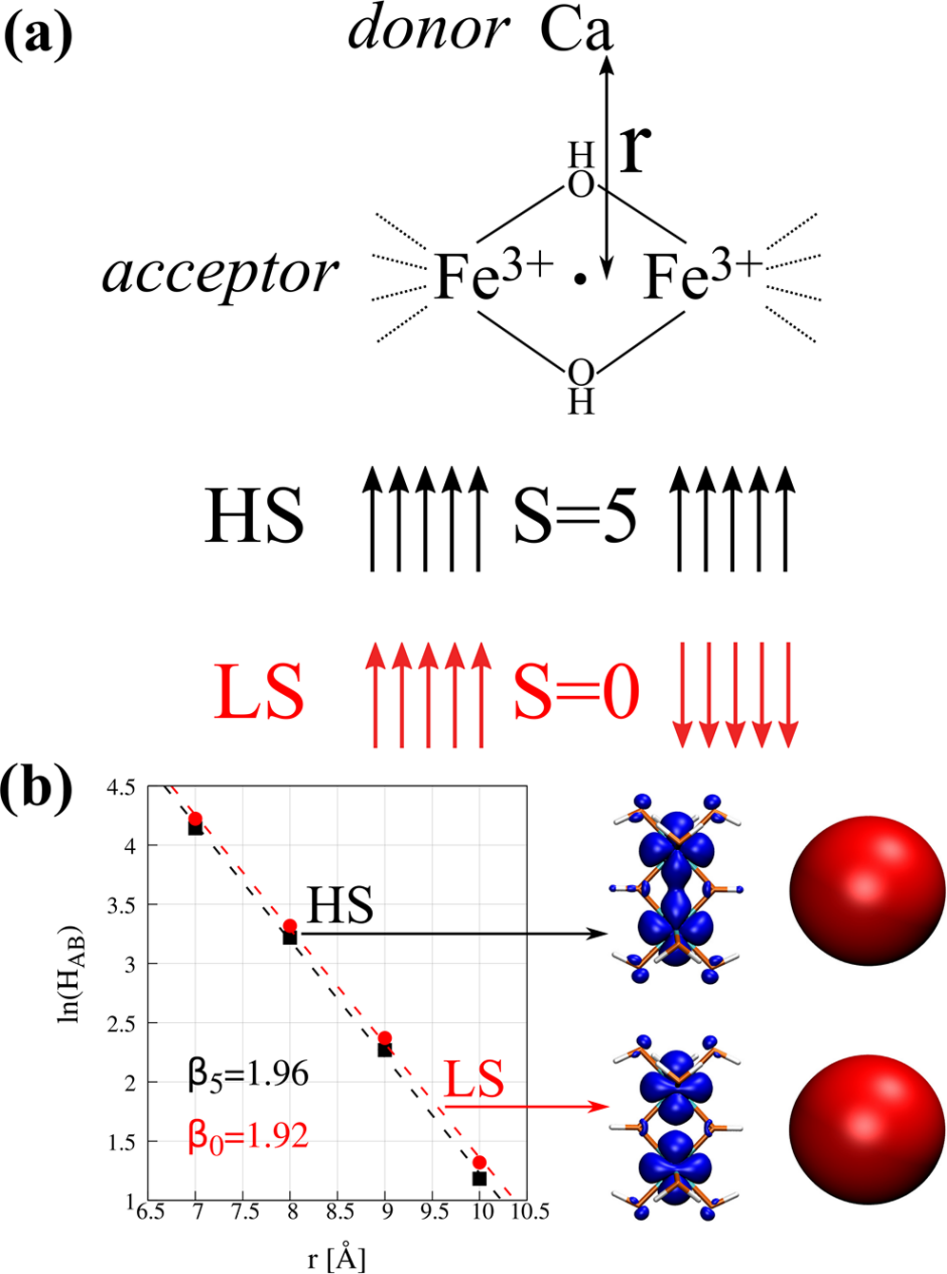
(a) Schematic representation of the model unsymmetric system for the electron transfer from the Ca atom to a system with small covalency (see Figure 3a). Diabatic distribution of 3d electrons in the high- (HS) and low-spin (LS) electronic states of the acceptor molecule is provided for the system before electron transfer. Panel (b) depicts the change of the *H*_AB_ with the distance *r* between donor and acceptor atoms. For each electronic state, the representative difference density plots are provided on the right (±0.0005 au).

The results for electronic coupling matrix element calculations for electron transfer from Ca to [Fe_2_(OH)_6_(H_2_O)_4_]^0^ are shown in Figure 4b. Interestingly, the couplings are very similar for both spin states of the system. The difference densities for both states are virtually identical so that the overlap between diabatic states is expected to be of similar magnitude for HS and LS spin states. It is somehow expected owing to the low covalency of the Fe-(μ-OH) bond as discussed above.

**Figure 5:**
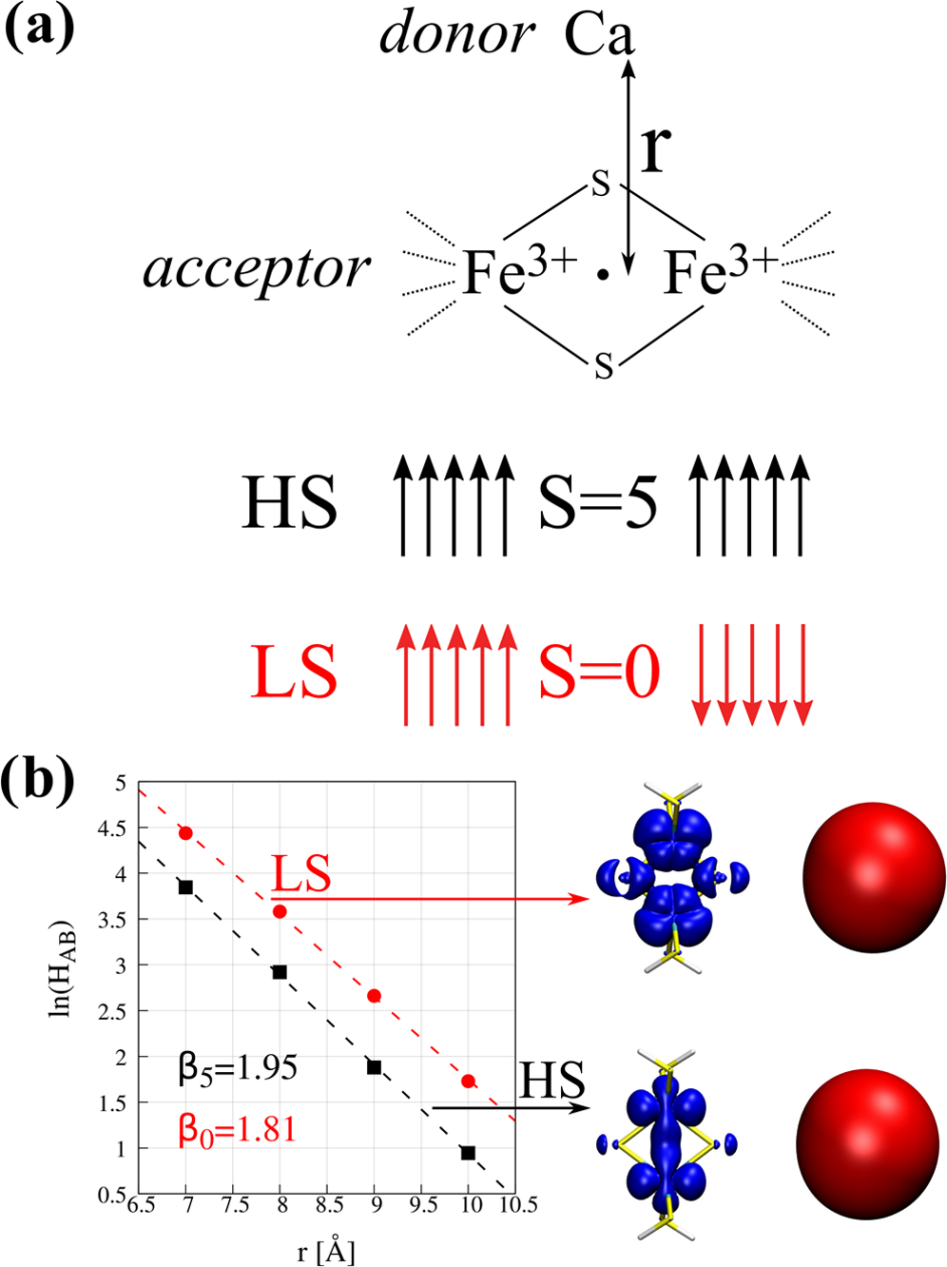
(a) Schematic representation of the model unsymmetric system for the electron transfer from the Ca atom to a system with significant covalency (see Figure 3b). Diabatic distribution of 3d electrons in the high- (HS) and low-spin (LS) electronic states of the acceptor molecule is provided for the system before electron transfer. Panel (b) depicts the change of the *H*_AB_ with the distance r between donor and acceptor atoms. For each electronic state, the representative difference density plots are provided on the right.

Contrary to the iron oxide model, the iron–sulfur complex displays a pronounced difference in the computed electronic coupling matrix elements at HS and LS spin states (Figure 5). We found the *H*_AB_ obtained at the LS state around twice as large as the HS state. The difference increases with the distance, which is reflected in a lower decay constant β in the LS spin state compared to that in the HS state (1.81 vs 1.95, respectively). Inspection of the density difference maps clearly shows the effect of the antiferromagnetic coupling in this system. We observed a more pronounced change of the electron density at the bridging sulfur atoms upon excitation for the LS state compared to that for the HS state. This is due to a larger overlap of the diabatic states in the former case. Moreover, the changes in the volumes around both iron atoms are also larger for the LS state, which can be understood as higher “flexibility” of this state that eases the electron addition.

## 4 Conclusions

The effect of a spin state of the donor and acceptor states is very diverse and highly dependent on the system under study. In principle, it can be traced back to the change of the electron density caused by spin-state alternation that affects the overlap integral between charge-localized (diabatic) states.

From a conceptual perspective, we classified the spin effects into three categories: orbital occupation, spin-dependent electron density, and unpaired density delocalization. The orbital occupancy captures the differential chemical nature and reactivity of the spin states of interest—the states can be characterized by very different *H*_AB_ values that may become close to each other once a structural distortion renders electronic densities of both states to be similar.

The spin-dependent electron density effect is connected with a more compact electron density cloud at lower spin states due to decreased exchange interactions between electrons. This accounts for significantly smaller *H*_AB_ values for antiferromagnetically coupled pairs compared to those for ferromagnetic pairs. In the absence of other factors, such as highly covalent ligands, the increased coupling may also be understood similarly to Girerd^93^ as the change of energy gap between the ground and first excited states at the top of the ET barrier that is proportional to the total spin of the system. Moreover, we have demonstrated that when the ET process takes place between high-spin centers, the effect of the spin can be easily incorporated with a spin-dependent factor. This observation may be explored when developing new approximate schemes for *H*_AB_ computations that rely on the linear dependency of the charge-transfer integral to the overlap between donor and acceptor molecules.

In molecular systems that feature highly covalent bonds, such as iron–sulfur clusters, the electronic density will be strongly affected by the ligand-to-metal interactions. The covalency effect increases the spatial extent of the electron density of the diabatic state in specific directions. With a model donor (calcium atom), we have shown that the ligand’s involvement significantly enhances electronic couplings in the low-spin state.

The findings presented in this work can be used to validate methods for electronic coupling matrix element calculations. Particular fields of applications are the systems that can undergo a spin-state change upon the action of an external stimulus, such as spin-crossover transition-metal complexes or materials featuring sites of the same metal in different spin states. Here, the desired property of a chosen method to compute *H*_AB_ should be the spin-state-independent error and this may be evaluated with model systems presented. All data required for comparison can be found in the Supporting Information.

Regarding the systems that have antiferromagnetic ground spin states, the presented work offers the first rigorous test set. These are typically modeled using DFT within the broken-symmetry approach.^24−26,31−33^ However, it is known that the final spin densities strongly depend on a particular choice of the density functional that in turn should influence the couplings to a larger degree.^94,95^

The couplings reported in Table 1 are generally the largest among all values computed in this study. Their magnitude points out that the applicability of eq 3 may be limited as such electron transfer may fall into an adiabatic regime^39,96,97^ and other rate expressions need to be used such as those by Zusman,^98^ Hartmann et al.,^99^ or Zhao and Liang.^100^ However, the relative magnitude of the electronic coupling (wrt other quantities that influence the electron-transfer process) is an important factor in the Robin–Day classification of mixed-valence compounds.^101,102^ In this context, the assessment of the accuracy of present methods for electronic coupling matrix element calculations may facilitate the design of novel materials incorporating mixed-valence compounds, e.g., single-molecule magnets where the high-spin stabilization is enhanced by the large double-exchange parameter *B* that in turn depends on the electronic coupling between magnetic centers.^103^ We also note that spin-dependent ET in multiheme proteins^104−106^ is a very actively developing field^107^ and our findings may facilitate the development of efficient simulation protocols in this case.

## Supporting Information Available

Electronic coupling values; active space orbitals and leading configurations; and Cartesian coordinates (PDF)
