# Supplementary material for: How the Donor/Acceptor Spin States Affect the Electronic Couplings in Molecular Charge-Transfer Processes?
Source: J Chem Theory Comput. 2021 Apr 8;17(5):2917–27. doi: 10.1021/acs.jctc.1c00126 (PMC8154369; doi:10.1021/acs.jctc.1c00126)
Supplement: Supplementary file 1 — ct1c00126_si_001.pdf [file ct1c00126_si_001.pdf]

# Supporting Information: How the donor/acceptor spin states affect the electronic couplings in molecular charge transfer processes?

A. Kubas*

*Institute of Physical Chemistry, Polish Academy of Sciences, Kasprzaka 44/52, 01-224 Warsaw, Poland*

E-mail: akubas@ichf.edu.pl

## Contents

**1 Electronic couplings values** **S-2**

1.1 $H_2 \cdots H_2^+$ . . . S-2

1.2 $Fe^{2+} \cdots Fe^{3+}$ . . . S-3

1.3 $[Fe_2(OH)_6(H_2O)_4]^0 \cdots Ca$ . . . S-4

1.4 $[Fe_2(S)_2(SH)_4]^{2-} \cdots Ca$ . . . S-4

**2 Active space orbitals and leading configurations** **S-5**

2.1 $Fe^{2+} \cdots Fe^{3+}$ . . . S-6

2.2 $[Fe_2(OH)_6(H_2O)_4]^0$ . . . S-7

2.3 $[Fe_2(S)_2(SH)_4]^{2-}$ . . . S-8

2.4 $[Fe_2(OH)_6(H_2O)_4]^0 \cdots Ca$ . . . S-9

2.5 $[Fe_2(S)_2(SH)_4]^{2-} \cdots Ca$ . . . . . . . . . . . . . . . . . . . . . . . . . . . . . . . . S-11

**3 Cartesian coordinates** **S-13**

3.1 $[Fe_2(OH)_6(H_2O)_4]^0 \cdots Ca$ . . . . . . . . . . . . . . . . . . . . . . . . . . . . . . . . S-13

3.2 $[Fe_2(S)_2(SH)_4]^{2-} \cdots Ca$ . . . . . . . . . . . . . . . . . . . . . . . . . . . . . . . . S-14

# 1 Electronic couplings values

The couplings $H_{AB}$ reported below are provided in meV. $S_{tot}$ corresponds to the total spin-state of the system under study. $\beta$ denotes the decay constant in the Eq. (9) in the main manuscript. The basis set used was ma-def2-TZVPP for Fe, O, S, Ca atoms and def2-TZVP for H atoms unless stated otherwise. Details of each method applied are provided in the main text.

## 1.1 $H_2 \cdots H_2^+$

Table S1: Electronic couplings obtained at MRCI+Q/aug-cc-pVTZ level for $H_2 \cdots H_2^+$ system at H-H distance of 0.74 Å.

| $d$ | $H_{AB}(S_{tot}=3/2)$ | $H_{AB}(S_{tot}=1/2)$ |
|---|---|---|
| 4.0 | 223.8 | 37.9 |
| 4.5 | 132.2 | 14.9 |
| 5.0 | 76.9 | 5.9 |
| 5.5 | 44.3 | 2.2 |
| 6.0 | 25.6 | 0.9 |
| $\beta$ | 2.18 | 3.78 |

Table S2: Electronic couplings obtained at MRCI+Q/aug-cc-pVTZ level for $H_2 \cdots H_2^+$ system at H-H distance of 2.50 Å.

| $d$ | $H_{AB}(S_{tot}=3/2)$ | $H_{AB}(S_{tot}=1/2)$ |
|---|---|---|
| 4.0 | 66.8 | 63.1 |
| 4.5 | 28.6 | 29.1 |
| 5.0 | 12.1 | 13.2 |
| 5.5 | 5.1 | 5.8 |
| 6.0 | 2.1 | 2.5 |
| $\beta$ | 3.46 | 3.22 |

Table S3: Electronic couplings for $H_2 \cdots H_2^+$ system at H-H distance of 0.75 Å and $d = 5.0$ calculated with various methods using aug-cc-pVTZ basis set. Diff. denotes difference between high-spin and low-spin couplings. Diff. RE stands for relative error of the difference w.r.t. full configuration interaction (FCI) method (in %).

| *Method* | $H_{AB}(S_{tot} = 3/2)$ | $H_{AB}(S_{tot} = 1/2)$ | Diff. | Diff. MRE |
|---|---|---|---|---|
| CASSCF(3,4) | 81.7 | 4.1 | 77.7 | 9.1 |
| CASSCF(3,20) | 77.0 | 5.1 | 71.9 | 1.1 |
| NEVPT2/CAS(3,4) | 78.5 | 4.9 | 73.6 | 2.5 |
| MRCI+Q/CAS(3,4) | 76.8 | 6.2 | 70.6 | 0.5 |
| MRCI+Q/CAS(3,20) | 76.9 | 5.9 | 71.0 | 0.1 |
| FCI | 76.9 | 5.8 | 71.1 | |

## 1.2 $Fe^{2+} \cdots Fe^{3+}$

Table S4: Electronic couplings for $Fe^{2+} \cdots Fe^{3+}$ system calculated at two spin-states using NEVPT2 method and two basis sets - unbracketed values denotes couplings obtained with ma-def2-TZVPP basis and values in parthenesis correspond to aug-cc-pVTZ basis.

| $d$ | $H_{AB}(S_{tot} = 9/2)$ | $H_{AB}(S_{tot} = 1/2)$ |
|---|---|---|
| 2.93 | 552.2 (555.4) | 108.5 (109.0) |
| 3.50 | 171.2 (176.2) | 34.3 (35.3) |
| 4.00 | 54.9 (57.4) | 11.0 (11.5) |
| 4.50 | 15.9 (17.3) | 3.1 (3.5) |
| 5.00 | 4.8 (5.1) | 1.0 (1.0) |
| $\beta$ | 4.61 (4.54) | 4.60 (4.50) |

## 1.3 $[Fe_2(OH)_6(H_2O)_4]^0 \cdots$ Ca

Table S5: Electronic couplings for $[Fe_2(OH)_6(H_2O)_4]^0 \cdots$ Ca system calculated at two spin-states using selected CASCI method.

| $d$ | $H_{AB}(S_{tot} = 5)$ | $H_{AB}(S_{tot} = 0)$ |
|---|---|---|
| 7.0 | 63.0 | 68.2 |
| 8.0 | 24.0 | 27.6 |
| 9.0 | 9.7 | 10.7 |
| 10.0 | 3.3 | 3.7 |
| $\beta$ | 1.96 | 1.92 |

## 1.4 $[Fe_2(S)_2(SH)_4]^{2-} \cdots$ Ca

Table S6: Electronic couplings for $[Fe_2(S)_2(SH)_4]^{2-} \cdots$ Ca system calculated at two spin-states using selected CASCI method.

| $d$ | $H_{AB}(S_{tot} = 5)$ | $H_{AB}(S_{tot} = 0)$ |
|---|---|---|
| 7.0 | 46.8 | 84.5 |
| 8.0 | 18.5 | 35.9 |
| 9.0 | 6.5 | 14.3 |
| 10.0 | 2.6 | 5.6 |
| $\beta$ | 1.95 | 1.81 |

## 2 Active space orbitals and leading configurations

This section contains isosurface plots ($\pm 0.03$) of the active space natural molecular orbitals (NMOs) obtained for all iron-containing systems at high- and low-spin states. These NMOs were obtained from converged state-averaged CASSCF calculations. Up to five leading configuration state functions (CSFs) of the ground and excited states (those used in coupling calculations) at respective spin states are provided below in each case. These follows the ORCA program convention *CSF coefficient* : *NMOs occupation vector*. For the calcium-containing systems, the CSFs were obtained at a modified CI level described in the main text.

## 2.1 $Fe^{2+} \cdots Fe^{3+}$

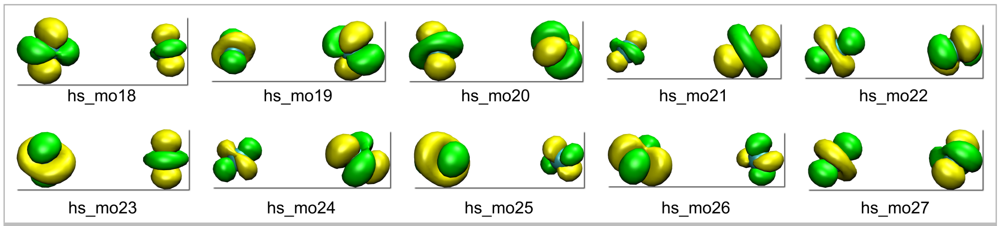


HS natural orbitals

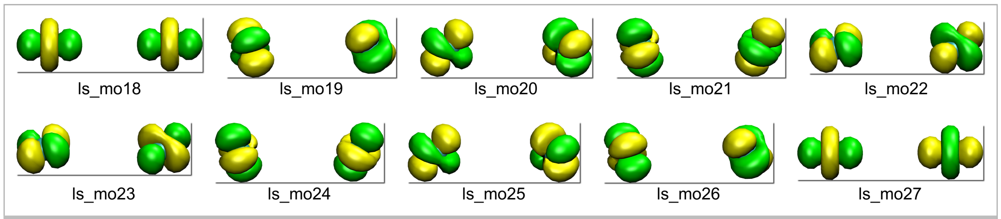


LS natural orbitals

### High-spin state

*Ground state:*

```
0.26344 : 1111111121
0.20967 : 1112111111
0.14630 : 2111111111
0.09504 : 1111112111
0.08855 : 1121111111
```

*Excited state:*

```
0.19916 : 1211111111
0.19622 : 1111121111
0.19373 : 1111112111
0.18162 : 1112111111
0.07827 : 2111111111
```

### Low-spin state

*Ground state:*

```
0.03751 : 2111111111
0.01874 : 1111021112
0.01874 : 1111201112
0.01874 : 1121111012
0.01874 : 1101111212
```

*Excited state:*

```
0.03748 : 1111111112
0.01876 : 2112110111
0.01876 : 2211111101
0.01876 : 2111201111
0.01876 : 2121111011
```

## 2.2 $[Fe_2(OH)_6(H_2O)_4]^0$

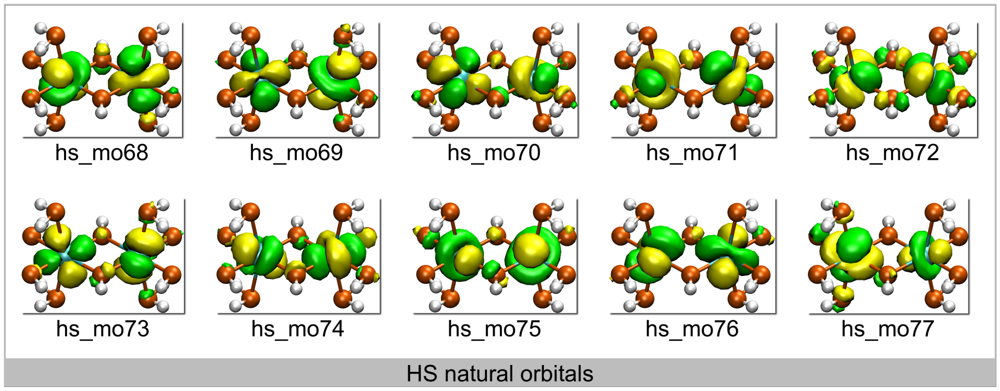


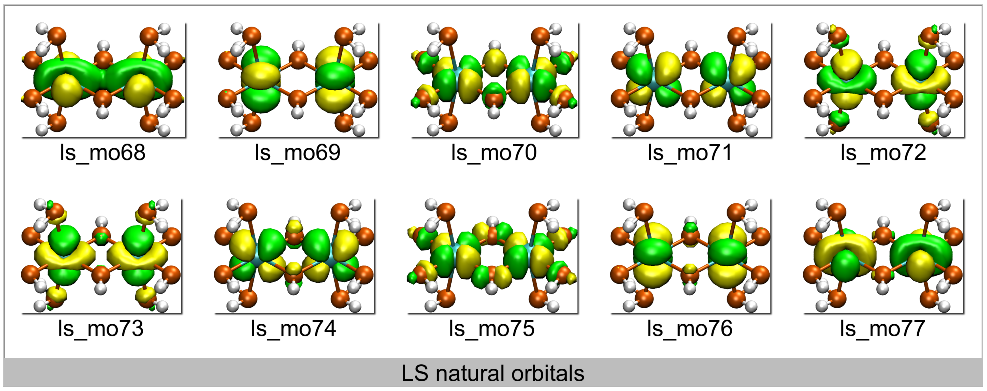


**High-spin state**

*Ground state:*

```
0.39065 : 1111111121
0.14230 : 1121111111
0.13318 : 1211111111
0.12239 : 1111111112
0.08607 : 1111111211
```

*Excited state:*

```
0.25730 : 1111112111
0.16339 : 1112111111
0.14980 : 1111121111
0.14465 : 2111111111
0.12865 : 1121111111
```

**Low-spin state**

*Ground state:*

```
0.03808 : 1112111111
0.02031 : 2111112110
0.01945 : 2212111100
0.01917 : 1211112101
0.01897 : 2122111010
```

*Excited state:*

```
0.03610 : 1111112111
0.02204 : 2112111110
0.02076 : 1212111101
0.02027 : 1122111011
0.01936 : 1112201111
```

## 2.3 $[Fe_2(S)_2(SH)_4]^{2-}$

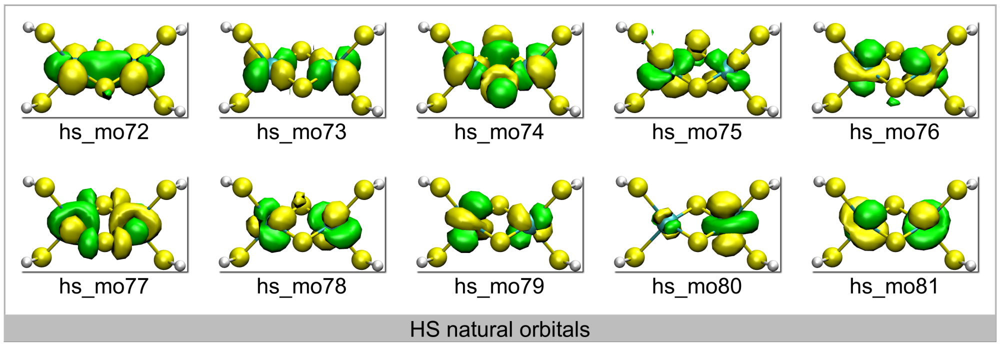

HS natural orbitals

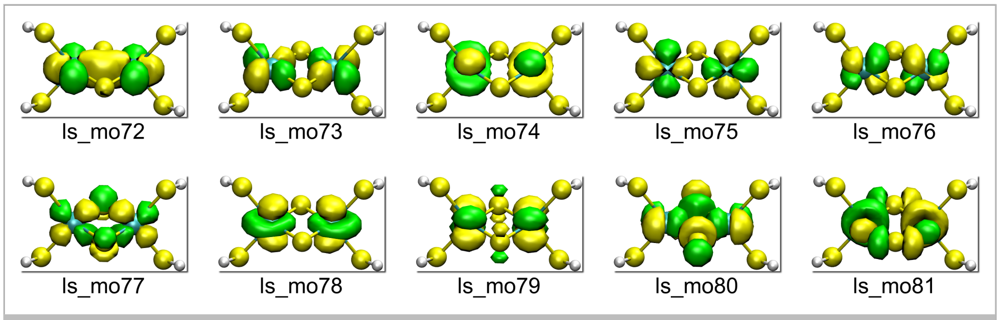

LS natural orbitals

### High-spin state

*Ground state:*

```
0.56148 : 1111121111
0.20328 : 1111111211
0.11481 : 1121111111
0.04529 : 1111211111
0.03652 : 1111111121
```

*Excited state:*

```
0.73456 : 2111111111
0.17963 : 1111111121
0.04918 : 1112111111
0.03103 : 1211111111
0.00441 : 1121111111
```

### Low-spin state

*Ground state:*

```
0.02556 : 2222200001
0.02073 : 2221111001
0.01943 : 2211201101
0.01900 : 2111111111
0.01798 : 2212110101
```

*Excited state:*

```
0.02406 : 2210112110
0.02349 : 2221111010
0.01789 : 2220022010
0.01726 : 2201111210
0.01675 : 1211111111
```

## 2.4 $[Fe_2(OH)_6(H_2O)_4]^0 \cdots Ca$

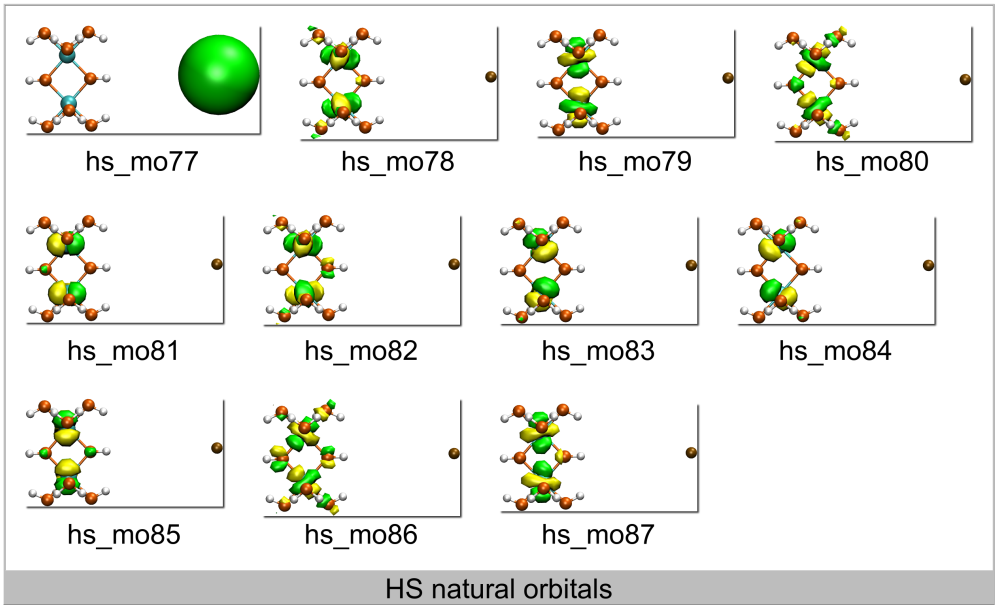


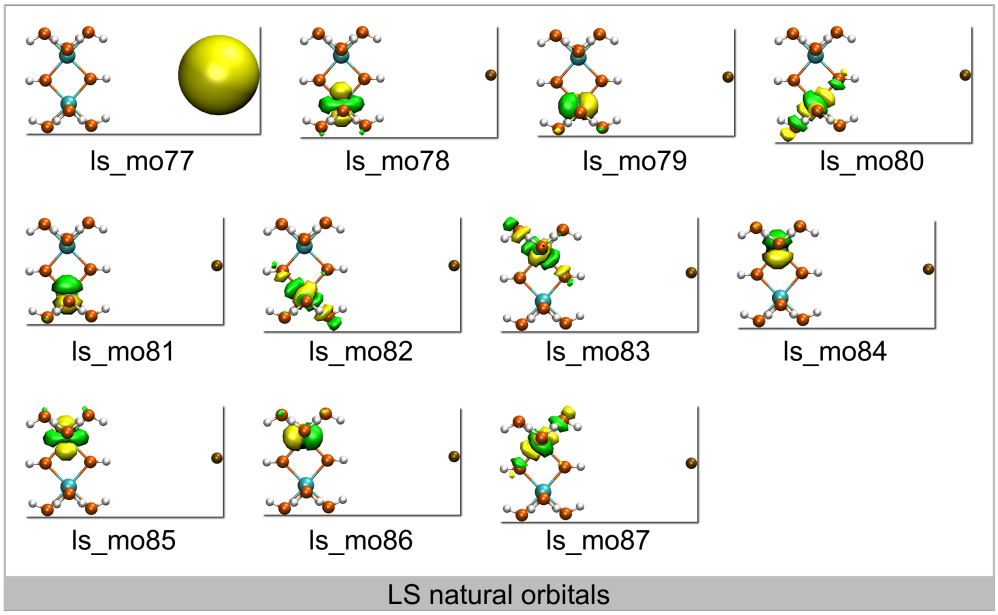

**High-spin state**

*Ground state:*

```
1.0000  : 21111111111
```

*Excited state:*

```
0.7139  : 11111111112
0.2725  : 12111111111
0.0136  : 11121111111
```

**Low-spin state**

*Ground state:*

```
0.9995  : 21111111111
```

*Excited state:*

```
0.2454  : 11111111112
0.2442  : 11111211111
0.2421  : 11111121111
0.2409  : 11121111111
0.0137  : 11111111211
```

## 2.5 $[Fe_2(S)_2(SH)_4]^{2-} \cdots Ca$

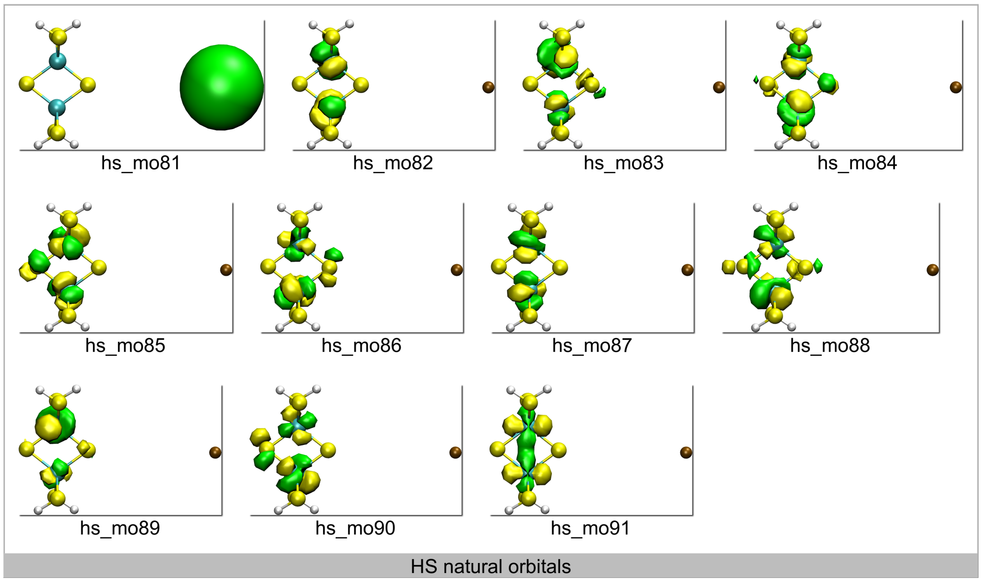


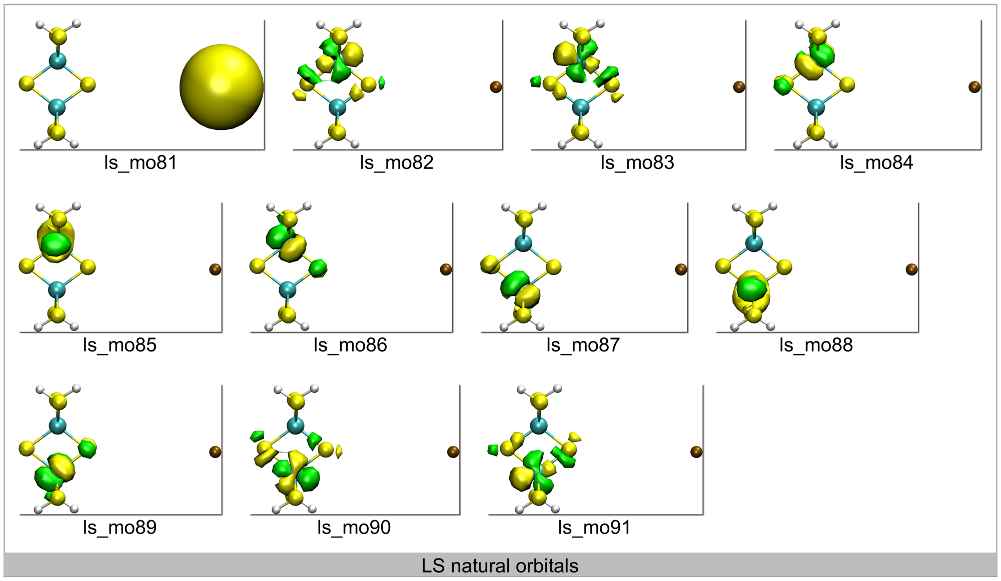

**High-spin state**

*Ground state:*

```
1.0000  : 21111111111
```

*Excited state:*

```
0.9983  : 12111111111
```

**Low-spin state**

*Ground state:*

```
0.9794  : 21111111111
```

*Excited state:*

```
0.2446  : 11111111121
0.2399  : 12111111111
0.2288  : 11211111111
0.2222  : 11111111112
0.0129  : 12011111121
```

# 3 Cartesian coordinates

The coordinates below [Å] correspond to iron clusters optimized with the method described in the manuscript main text. The distance between cluster center the and calcium atom ($d$) was varied between 7 and 10 Å. Here, we selected $d$ = 7 Å for demonstration purposes but other geometries can be easily obtained by changing $x$ coordinate of the calcium atom.

## 3.1 $[Fe_2(OH)_6(H_2O)_4]^0 \cdots Ca$

```
Fe       0.000000000      0.000000000      2.927441600
O       -1.323463300      0.000000000      4.282291200
O       -1.354544500      0.000000000      1.463720800
O        1.354544500      0.000000000      1.463720800
O        1.323463300      0.000000000      4.282291200
O        0.000000000      2.120914900      3.309244600
O        0.000000000     -2.120914900      3.309244600
H       -0.765778400     -1.951306900      3.913945500
H       -2.318983500      0.000000000      1.463720800
H        2.318983500      0.000000000      1.463720800
H        0.765778400      1.951306900      3.913945500
H       -2.203279700      0.000000000      3.861868900
H        2.203279700      0.000000000      3.861868900
H       -0.765778400      1.951306900      3.913945500
H        0.765778400     -1.951306900      3.913945500
Fe       0.000000000      0.000000000      0.000000000
O       -1.323463300      0.000000000     -1.354849600
O        1.323463300      0.000000000     -1.354849600
O        0.000000000      2.120914900     -0.381803000
O        0.000000000     -2.120914900     -0.381803000
H       -0.765778400     -1.951306900     -0.986503900
H        2.203279700      0.000000000     -0.934427300
H        0.765778400      1.951306900     -0.986503900
H       -2.203279700      0.000000000     -0.934427300
H       -0.765778400      1.951306900     -0.986503900
H        0.765778400     -1.951306900     -0.986503900
Ca       7.000000000      0.000000000      1.465000000
```

### 3.2 $[Fe_2(S)_2(SH)_4]^{2-} \cdots Ca$

| | | | |
|---|---|---|---|
| Fe | 0.000000000 | 0.000000000 | 0.000000000 |
| Fe | 0.000000000 | 0.000000000 | 2.653060532 |
| S | 1.744809167 | 0.000000000 | 1.326420961 |
| S | -1.743591980 | -0.004224920 | 1.325883835 |
| H | -0.941352652 | 1.566496093 | -2.088481471 |
| S | -0.187187500 | -1.866175950 | 4.061574977 |
| H | 0.926649921 | -1.542715227 | 4.761705251 |
| S | 0.183150094 | 1.871635544 | 4.050079328 |
| H | -0.932103220 | 1.556129612 | 4.751612727 |
| S | -0.182382487 | -1.870039272 | -1.403750701 |
| H | 0.938254486 | -1.553880977 | -2.096315271 |
| S | 0.180226621 | 1.874040461 | -1.393517551 |
| Ca | 7.000000000 | 0.000000000 | 1.326000000 |
